# The impact of baseline [−2]proPSA-related indices on the prediction of pathological reclassification at 1 year during active surveillance for low-risk prostate cancer: the Japanese multicenter study cohort

**DOI:** 10.1007/s00432-013-1566-2

**Published:** 2013-12-19

**Authors:** Hiromi Hirama, Mikio Sugimoto, Kazuto Ito, Taizo Shiraishi, Yoshiyuki Kakehi

**Affiliations:** 1 Department of Urology, Kagawa University Faculty of Medicine, 1750-1 Ikenobe, Miki-cho, Kita-gun, Kagawa 761-0793 Japan; 2 Department of Urology, Gunma University Graduate School of Medicine, 3-39-22, Showa-machi, Maebashi, Gunma 371-8511 Japan; 3 Department of Pathologic Oncology, Mie University Graduate School of Medicine, 2-174 Edobashi, Tsu, Mie 514-8507 Japan

**Keywords:** [−2]proPSA, Active surveillance, Japanese cohort, Low-risk prostate cancer, Pathological reclassification

## Abstract

**Purpose:**

Active surveillance (AS) is one potential solution to avoiding the overtreatment of favorable prostate cancer. By handling the AS strategy more safely, tumor aggressiveness may be evaluated more accurately. The aim of the present study was to evaluate the predictive impact of baseline prostate-specific antigen (PSA) isoform [−2]proPSA (p2PSA)-related indices on the pathological reclassification at 1 year during an AS program.

**Methods:**

Between 2002 and 2003, 134 males diagnosed with low-risk prostate cancer were registered in the Japanese multicenter study cohort as candidates for AS, and 118 (88 %) males actually proceeded to AS. Of the 118 patients, the 67 that underwent protocol biopsy at 1 year after beginning AS were enrolled in the present study. The predictive significance of various baseline clinicopathological features and p2PSA-related indices on pathological reclassification at 1 year after beginning AS were investigated.

**Results:**

The pathological reclassification rate was 37.3 %. According to the univariate analysis, prostate volume (*p* = 0.049), number of biopsy cores (*p* = 0.047), percentage of positive biopsy cores (*p* = 0.023), p2PSA to free PSA ratio (%p2PSA) (*p* = 0.003) and prostate health index (phi) (*p* = 0.010) at baseline were significantly different between the reclassification and non-reclassification groups. By multivariate logistic regression analysis, baseline %p2PSA (*p* = 0.008) and phi (*p* = 0.008) were the only independent predictive factors for pathological upgrade at 1 year after AS commencement.

**Conclusions:**

Baseline %p2PSA and phi may predict the pathological reclassification at 1 year after starting AS, which could be due to the under detection of clinically significant prostate cancer at AS enrollment.

## Introduction

PSA-based screening was found to benefit survival in males ages 50 to 69 in the European Randomized Study of Screening for Prostate Cancer (ERSPC) (Schroder et al. 2012) and the Goteborg prospective randomized study (Hugosson et al. 2010). This study further demonstrated that one male per 1,055 screened, primarily at 4-year intervals (Schroder et al. 2012), and one male per 293 screened at 2-year intervals (Hugosson et al. 2010) would not suffer prostate cancer-related death over a decade. To avert prostate cancer over a lifetime, however, the survival benefit must be much greater. Issues of over-detection and subsequent overtreatment have emerged as serious drawbacks of PSA-based screening. Updated ERSPC data assumed that 37 radical treatments were required to prevent one death from prostate cancer (Schroder et al. 2012). Any radical treatment has a risk of decreasing QOL, thus AS may have an important role in decreasing the frequency of over-treatments throughout life. Candidates for AS are usually selected according to pretreatment pathological features of prostate biopsies and initial PSA values. Previous retrospective studies have demonstrated that a substantial number of patients who qualified for AS but underwent immediate radical prostatectomy (RP) were upgraded by the results of whole-prostate specimens (Behbahani et al. 2012; Iremashvili et al. 2012). Since 20–30 % of the initial biopsy underestimation is unavoidable, most AS protocols include strict PSA kinetics monitoring. Conversely, unreliable PSA and PSA kinetics readings during AS have been reported (Sugimoto et al. 2010; Ross et al. 2010; NCCN guidelines TM version 2013). Therefore, periodic repeat biopsies for patients with intermediate PSA doubling time (PSADT) are recommended for detection of aggressive tumors during AS. Such precautions may help to avoid delaying radical treatment.

To compensate for the unreliability of PSA, PSA kinetics or prostate biopsies, proPSA (Peter et al. 2001; Mikolajczyk et al. 2001) may be a new candidate biomarker employable at enrollment or AS monitoring, as p2PSA-related indices have high specificity not only for prostate cancer but also for other high-grade and high-volume tumors (Jansen et al. 2010; Catalona et al. 2011; Ito et al. 2012; Guazzoni et al. 2012). The aim of the present retrospective study was to evaluate the predictive significance of p2PSA-related indices on the likelihood of pathological reclassification at the 1 year protocol biopsy during AS in the Japanese multicenter prospective AS cohort.

## Materials and methods

Seven cancer center hospitals and six university hospitals in Japan participated in the multicenter prospective one-arm cohort study of AS. The institutional review board of each participating institution approved the study protocol. Participants in the AS study were subject to the following inclusion criteria: (1) stage T1cN0M0 (2) age 50–80 (3) serum PSA ≤ 20 ng/ml (4) 1 or 2 positive cores per 6–12 systematic biopsy cores (5) Gleason score (GS) ≤6 and (6) maximum cancer involvement in positive cores of ≤50 %. All biopsy specimens at entry and during AS were diagnosed by one central urologic pathologist (T.S.).

Between January 2002 and December 2003, 134 patients who met the inclusion criteria were registered after providing written informed consent. Of the 134 patients, 118 (88.1 %) actually proceeded to AS, while the remaining 16 patients preferred to undergo immediate radical treatments. Patients who opted for the AS program had PSA measurements every 2 months for the initial 6 months, with follow-ups every 3 months thereafter. Serum samples were collected and stored at −80 °C at each PSA measurement, according to the study protocol. Prostate biopsy was recommended for patients who remained on AS for 1 year. PSADT shorter than 2 years, or pathological reclassification (GS 7 or greater, more than two positive biopsy cores or more than 50 % cancer involvement of any biopsy core) upon the 1 year prostate biopsy was trigger for curative intervention.

One year after participating in the AS study, 15 (12.7 %) patients discontinued AS. Elimination reasons were PSADT <2 years for seven patients, patient preference for five patients, one patient underwent RP due to difficult voiding, one patient transferred to another hospital, and one patient was lost during follow-up. Of the remaining 103 patients who continued AS, 67 (65.0 %) patients underwent repeat biopsy and 36 (35.0 %) did not at 1 year after commencing AS.

Total PSA, free PSA and p2PSA were measured in serum samples collected upon entry into the study using the UniCel DxI800 Immunoassay System analyzer (Beckman Coulter Inc., Brea, CA, USA). Hybritech calibrators (Beckman Coulter Inc., Brea, CA, USA) were used for total PSA and free PSA assays. Various clinicopathological features upon AS entry and baseline free to total PSA ratio (%free PSA) (base model), %p2PSA and phi (extended model) were investigated in terms of their predictive value for prostate biopsy pathological reclassification. Phi was calculated using the following formula: (p2PSA/free PSA) × √total PSA.

All statistical analyses were performed using the SPSS ver. 20.0 (SPSS) or Stat Flex ver. 6.0 software package. Significance was calculated using the Mann-Whitney *U* test for continuous variables and the chi-square test for categorical variables. A *p* value of <0.05 was considered to indicate statistical significance. Cutoffs of the above clinicopathological factors for multivariable analyses were explored by separating patients into binary, tertiary, quartiles or quintiles in order to establish more significant and meticulous separation. If two adjacent subgroups were considered to have an equal predictive value, they were combined.

A stepwise multiple logistic regression analysis was used to determine independent significant predictive factors, in which all clinicopathological factors were handled as categorical variables.

## Results

The median age of the 67 patients at the time of enrollment was 70 years (ranging from 55 to 79). Pathological reclassification 1 year after beginning AS was identified in 37.3 % (25/67) patients. The reasons for pathological reclassification in 25 of the patients are shown in Table 1. Among these 25 patients, 15 (60.0 %) had only one upgrading factor and 10 (40.0 %) had any combination of two or three upgrading factors. Table 2 displays comparisons of the baseline clinicopathological features of males with or without pathological reclassification. Prostate volume (*p* = 0.049), biopsy core number (*p* = 0.047) and the percentage of positive cores (*p* = 0.023) at baseline were significantly different between the reclassification and non-reclassification groups, while other parameters did not differ significantly.

**Table 1:** Result of repeat biopsy at 1 year after entering the AS study

	No. of patients (%)
Reclassification	25 (37.3)
Only no. of positive cores >2	10 (14.9)
Only Gleason score (GS) >6	4 (6.0)
Only % of maximum cancer involvement ≥50 %	1 (1.5)
GS >6 and no. of positive cores >2	3 (4.5)
GS >6 and % of maximum cancer involvement ≥50 %	2 (3.0)
GS >6, no. of positive cores >2 and % of maximum cancer involvement ≥50 %	5 (7.5)
Non-reclassification	42 (62.7)
No cancer	23 (34.3)

% of maximum cancer involvement; occupancy of maximum cancer length (mm)/total core length (mm) in positive biopsy specimen × 100 (%)

**Table 2:** Differences in the baseline clinicopathological features between the reclassification and non-reclassification groups

	Reclassification group (*n* = 25)	Non-reclassification group (*n* = 42)	*p* value
Age			
Median (range)	69 (60–78)	70 (55–79)	0.682*
Mean ± SD	69.48 ± 4.80	69.57 ± 4.96	
Prostate volume (cc)			
Median (range)	31.3 (8–102.8)	40.3 (15–97.8)	0.049*
Mean ± SD	41.08 ± 19.34	43.99 ± 18.89	
Total PSA (ng/ml)			
Median (range)	6.4 (3.9–17.0)	6.05 (2.9–13.0)	0.831*
Mean ± SD	7.31 ± 3.58	6.92 ± 2.80	
PSAD (ng/ml/cc)			
Median (range)	0.18 (0.9–0.84)	0.15 (0.06–0.50)	0.089*
Mean ± SD	0.25 ± 0.20	0.18 ± 0.11	
Gleason score			
5 (%)	3 (12.0 %)	5 (11.9 %)	1.000**
6 (%)	22 (88.0 %)	37 (88.1 %)	
No. of positive cores			
1 (%)	16 (64.0 %)	35 (83.3 %)	0.073**
2 (%)	9 (34.0 %)	7 (16.7 %)	
No. of biopsy core taken			
6–8 (%)	19 (76.0 %)	22 (52.4 %)	0.047**
9–12 (%)	6 (24.0 %)	20 (47.6 %)	
% of positive cores			
Median (range)	16.7 (8.3–33.3)	12.5 (8.3–33.3)	0.023*
Mean ± SD	17.70 ± 7.65	14.09 ± 6.66	
10.0 or less (%)	4 (16.0 %)	16 (38.1 %)	0.048**
Greater than 10.0 (%)	21 (84.0 %)	26 (61.9 %)	
% of maximum cancer involvement (%)			
Median (range)	10 (3.6–30)	11.75 (2.1–42.9)	0.943*
Mean ± SD	14.02 ± 9.11	13.67 ± 8.88	

% of maximum cancer involvement; occupancy of maximum cancer length (mm)/total core length (mm) in positive biopsy specimen × 100 (%)

* Mann-Whitney *U* test, ** Chi-square test

Table 3 shows comparisons of baseline %free PSA, %p2PSA and phi between the reclassification and non-reclassification groups. Baseline %free PSA, %p2PSA and phi were handled as both continuous and categorical variables. There were no significant differences in %free PSA between the two groups when used as continuous (*p* = 0.805) or categorical (*p* = 0.462) variables. Conversely, %p2PSA and phi in the reclassification group were significantly higher than those in the non-reclassification group when used as continuous or categorical variables (%p2PSA, *p* = 0.003 and *p* = 0.013, respectively; phi, *p* = 0.010 and *p* = 0.007, respectively).

**Table 3:** Differences in the PSA-related indices between the reclassification and non-reclassification groups

	Reclassification group (*n* = 25)	Non-reclassification group (*n* = 42)	*p* value
%free PSA			
Median (range)	15.96 (4.55–39.67)	14.70 (5.86–31.68)	0.805*
Mean ± SD	16.67 ± 7.62	16.15 ± 6.86	
15.96 or less (%)	12 (48.0 %)	22 (52.4 %)	0.462**
>15.96 (%)	13 (52.0 %)	20 (47.6 %)	
%p2PSA			
Median (range)	2.44 (1.13–6.21)	1.88 (0.78–6.63)	0.003*
Mean ± SD	2.75 ± 1.20	2.11 ± 1.12	
1.51 or less (%)	2 (8.0 %)	11 (26.2 %)	0.013** (−1.51 vs. 1.51–2.25 vs. 2.25–2.93 vs. 2.93–)
1.51 to 2.25 (%)	7 (28.0 %)	20 (47.6 %)	0.008** (−1.15 vs. 1.15–2.25 vs. 2.25−)
2.25 to 2.93 (%)	7 (28.0 %)	7 (16.7 %)	0.014** (−1.15 vs. 1.15–2.93 vs. 2.93−)
>2.93 (%)	9 (36.0 %)	4 (9.5 %)	0.006** (−2.25 vs. 2.25–2.93 vs. 2.93−)
			0.063** (−1.51 vs. 1.51−)
			0.003** (−2.25 vs. 2.25−)
			0.011** (−2.93 vs. 2.93−)
Phi			
Median (range)	60.3 (32.9–141.3)	47.8 (23.8–199.6)	0.010*
Mean ± SD	72.4 ± 31.3	55.7 ± 31.5	
43.2 or less (%)	3 (12.0 %)	14 (33.3 %)	0.007** (−43.2 vs. 43.2–76.3 vs. 76.3–)
43.2 to 76.3 (%)	11 (44.0 %)	23 (54.8 %)	0.046** (−43.2 vs. 43.2–)
>76.3 (%)	11 (44.0 %)	5 (11.9 %)	0.004** (−76.3 vs. 76.3–)

* Mann-Whitney *U* test, ** Chi-square test

**Fig. 1:**
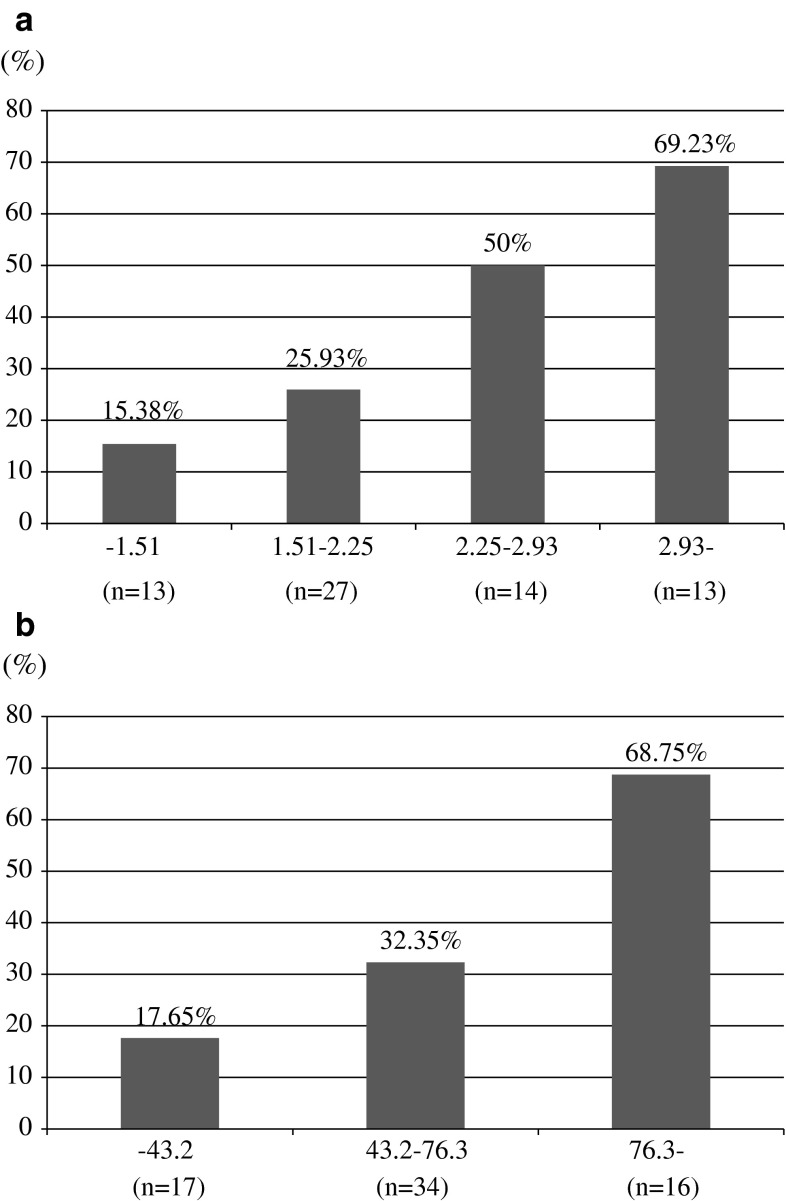
**a** Likelihood of pathological reclassification at 1 year after entering AS by categorized  %p2PSA values. **b** Likelihood of pathological reclassification at 1 year after entering AS by categorized phi values

Table 4 shows the results of multivariate logistic regression analyses using a base model that included age, prostate volume, percentage of positive biopsy core, maximum cancer involvement and %free PSA. Only prostate volume was a significant predictive factor for pathological reclassification at 1 year following AS in the base model. However, if p2PSA-related indices were included in the base model, %p2PSA (*p* = 0.008) and phi (*p* = 0.008) were the only independent predictive factors for pathological reclassification at 1 year after entering the AS study. Figure 1a shows that the biopsy reclassification rate increased with %p2PSA levels. The biopsy reclassification percentage at 1 year increased from 15.38 % in the lowest group (%p2PSA 1.51 % or less) to 69.23 % in the highest group (%p2PSA greater than 2.93 %). The biopsy reclassification rate also increased with phi levels (Fig. 1b). These results demonstrate a strong correlation of baseline %p2PSA and phi with the likelihood of pathological reclassification at 1 year after beginning AS.

**Table 4:** Multivariate analyses using base model and extended model including  %p2PSA or phi on the base model

	Base model	Base model + %p2PSA	Base model + phi
Age	0.465 (0.148–1.462)/0.190	0.480 (0.145–1.591)/0.231	0.420 (0.124–1.423)/0.164
Prostate volume	4.304 (1.121–16.522)/0.033	2.859 (0.681–12.004)/0.151	3.235 (0.767–13.641)/0.110
% of positive cores	2.747 (0.751–10.044)/0.127	3.539 (0.809–15.476)/0.093	3.264 (0.757–14.071)/0.113
% of maximum cancer involvement	0.954 (0.320–2.851)/0.933	0.962 (0.299–3.097)/0.948	1.026 (0.318–3.310)/0.966
%free PSA	0.604 (0.192–1.904)/0.390	0.468 (0.134–1.632)/0.234	0.384 (0.105–1.401)/0.147
%p2PSA	–	2.356 (1.252–4.435)/0.008	–
Phi	–	–	3.650 (1.408–9.461)/0.008

odds ratio (95 % confidence interval)/*p* value

Age (years): 70 or less (0), greater than 70 (1)

Prostate volume (cc): 47.0 or less (1), greater than 47.0 (0)

Percentage of positive cores (%): 10.0 or less (0), greater than 10.0 (1)

Maximum cancer involvement (mm) 11.5 or less (0), greater than 11.5 (1)

%free PSA: 15.96 or less (1), greater than 15.96 (0)

%p2PSA: 1.51 or less (0), 1.51–2.25 (1), 2.25–2.93 (2), greater than 2.93 (3)

Phi: 43.2 or less (0), 43.2–76.3 (1), greater than 76.3 (2)

% of maximum cancer involvement; occupancy of maximum cancer length (mm)/total core length (mm) in positive biopsy specimen × 100 (%)

## Discussion

In the present study, there was a significant association of baseline %p2PSA and phi with pathological reclassification at a 1 year repeat biopsy. These results suggest that p2PSA-related parameters at diagnosis may be predictive of an underestimation of clinically significant prostate cancer at AS enrollment.

Some studies have shown the usefulness of proPSA-related parameters in prostate cancer detection. Jansen et al. reported that phi had a higher AUC for prostate cancer detection than either total PSA or %free PSA in two European populations (Jansen et al. 2010). Catalona et al. similarly reported that the AUC for phi was higher than those of total PSA or %fPSA in a multicenter study that included 892 males. They demonstrated that males with phi 55.0 or higher had a 4.7-fold greater risk of prostate cancer compared to those with a phi of less than 25.0 (Catalona et al. 2011). They further showed that the use of phi could prevent 28 and 33 % of unnecessary biopsies while maintaining 95 and 90 % sensitivity, respectively. Moreover, Ito et al. recently demonstrated a very high specificity of prostate volume-adjusted p2PSA-related indices at a sensitivity of 90 % in a Japanese cohort (Ito et al. 2012). With respect to the relationships between grade and cancer volume and p2PSA-related parameters, Ito et al. also demonstrated significant relationships between the level of phi and prostate volume-adjusted p2PSA-related indices with GS and cancer occupancy in biopsy specimens. Furthermore, Guazzoni et al. reported that %p2PSA and phi significantly discriminated males with pT3 disease, pathologic Gleason sum ≥7 and Gleason sum upgrading based on RP specimens (Guazzoni et al. 2012).

Regarding the predictive power during AS of p2PSA and p2PSA-related parameters for reclassification at repeat biopsy, Tosoian and colleagues recently reported that p2PSA and p2PSA-related parameters were predictive of reclassification in very low-risk patients who underwent AS (Tosoian et al. 2012). They showed that baseline and longitudinal changes in the %free PSA, %p2PSA, p2PSA/%free PSA and phi values were associated with reclassification at repeat biopsies. These results are comparable to those obtained in the present study despite the different ethnicities examined (primarily Caucasians versus Asians). The inclusion criteria in both cohorts were similar, but ours were less stringent, in that initial PSA could be up to 20 ng/ml and there was no restriction on PSA density. The present study focused on the likelihood of reclassification at a 1 year repeat biopsy while Tosoian et al. assessed reclassification during relatively long-term follow-up (a median of 4.3 years).

Most reclassifications at a 1 year repeat biopsy may not be due to disease progression, but to underestimation at initial biopsy. Our results demonstrate the high diagnostic ability of p2PSA-related parameters for discriminating candidates for AS with non-significant prostate cancer from those with significant cancer. Classic clinicopathological factors, including prostate volume, which were significantly predictive of biopsy reclassification by the base model, became less significant when %p2PSA or phi was analyzed by multivariate logistic regression. Thus, %p2PSA and phi could be independent significant factors when divided into quartiles and quintiles, respectively. This suggests that %p2PSA and phi are useful markers. Use of these markers may reduce the incidence of underestimation at initial diagnosis, enabling more accurate selection of suitable candidates for AS.

This retrospective study had several limitations. First, a median of eight biopsy cores were obtained (range, 6–12), which is relatively few compared to contemporary practice. Most Japanese institutions performed eight-core biopsies during the study period. Therefore, the relatively high reclassification ratio in the present study may be due to the fewer biopsy cores taken. The likelihood of pathological reclassification was significantly higher in patients from whom 6 to 8 biopsy cores were taken, compared to those from whom 9 to 12 cores were taken at study entry (Chi-square test *p* = 0.047). This suggests that an immediate second repeat multiple-core biopsy should be performed prior to AS enrollment to judge candidates based on eight or fewer biopsy cores. Second, we enrolled patients with a relatively high initial PSA level (10–20 ng/ml). However, there was no significant difference in total PSA between the reclassification and non-reclassification groups. Furthermore, there was no significant difference in reclassification rates between patients with PSA ≤ 10 ng/ml and those with PSA in the reflex range of 10–20 ng/ml. Including PSA values of 10–20 ng/ml may thus not affect the core results of the present study. Finally, the acceptance rates of repeat biopsies were low, at 65.0 %. However, there were no significant differences in the baseline clinicopathological findings among the following three groups: 67 patients that underwent repeat biopsy, 36 patients that refused repeat biopsy and 15 patients that discontinued AS within 1 year (data not shown).

In conclusion, baseline %p2PSA and phi were the only independent factors predictive of the likelihood of pathological reclassification at 1 year after AS commencement. This may be due to the under detection of clinically significant prostate cancer at the time of AS enrollment. However, baseline total PSA, %free PSA and classical clinicopathological findings were not significantly associated with biopsy reclassification. Parameters including p2PSA might be promising and supplemental biomarkers of tumor aggressiveness and burden. These p2PSA-related indices may enhance the safety of the AS program. In the future, longitudinal p2PSA-related indices monitoring during AS will be valuable.
